# Periodontal Pathobionts and Respiratory Diseases: Mechanisms of Interaction and Implications for Interdisciplinary Care

**DOI:** 10.3390/biomedicines13071741

**Published:** 2025-07-16

**Authors:** Byeongguk Kim, Nana Han

**Affiliations:** 1 Department of Stomatology, Tongji Hospital, Tongji Medical College, Huazhong University of Science and Technology, Wuhan 430030, China; kimbg0226@gmail.com; 2 School of Stomatology, Tongji Medical College, Huazhong University of Science and Technology, Wuhan 430030, China; 3 Hubei Province Key Laboratory of Oral and Maxillofacial Development and Regeneration, Wuhan 430022, China

**Keywords:** periodontitis, respiratory diseases, periodontal pathogen, periodontal treatment, interdisciplinary diseases

## Abstract

Periodontitis is a prevalent chronic inflammatory disease that has been increasingly recognized for its systemic impacts, including its connection to respiratory diseases such as pneumonia, chronic obstructive pulmonary disease (COPD), Obstructive Sleep Apnea (OSA), asthma, lung cancer, and COVID-19. This review explores the potential role of periodontal pathobionts, particularly *Porphyromonas gingivalis* (Pg), *Treponema denticola* (Td), *Fusobacterium nucleatum* (Fn), *Aggregatibacter actinomycetemcomitans* (Aa), and *Tannerella forsythia* (Tf), in respiratory health. These pathobionts contribute to respiratory diseases by facilitating pathogen adhesion, inducing epithelial cell apoptosis, and promoting inflammation. The review also highlights the beneficial effects of periodontal treatment in reducing pathobiont burden and systemic inflammation, thereby mitigating the risk of respiratory complications. This interdisciplinary approach underscores the need to consider oral health as a critical component in managing and preventing respiratory diseases, with future research needed to further clarify these associations and develop targeted interventions.

## 1. Introduction

### 1.1. Background of Periodontal Microorganisms and Periodontitis

Periodontitis is a chronic inflammatory and destructive disease of the periodontal tissues, caused by a combination of bacteria, host immunological responses, and other contributing factors [1]. It is prevalent globally and remains the primary etiological driver of adult tooth loss, affecting roughly 12% of the global adult population with severe periodontal disease [1,2]. The bacterial composition in periodontal pockets plays a crucial role in disease progression. Bacterial components like lipopolysaccharides initiate local and systemic inflammation, contributing to tissue destruction [3]. Recent advances in periodontal microbiology have demonstrated a complex interaction between oral microbiota and systemic health, with specific associations between periodontitis and a variety of systemic conditions [3,4,5].

### 1.2. Close Relationship Between Periodontitis and Systemic Diseases

A growing body of evidence has identified a close relationship between periodontitis and systemic diseases, such as cardiovascular diseases, diabetes mellitus, rheumatoid arthritis, and neurodegenerative disorders [6,7,8]. For example, *Porphyromonas gingivalis* and other periodontal pathogens have been detected in atherosclerotic plaques, implicating them in cardiovascular disease [7,8]. Additionally, bacteria such as *Actinobacillus actinomycetemcomitans* have been detected in half of the analyzed carotid endarterectomy specimens from clinically confirmed atherosclerotic patients, supporting the hypothesis that periodontal pathogens contribute to systemic bacteremia [9]. Accumulating evidence has demonstrated a periodontal–systemic axis manifesting as neurocognitive decline (including Alzheimer’s disease), gestational complications (preterm birth/low birth weight), and autoimmune arthritis pathogenesis [10,11]. Mechanistic studies further reveal that chronic periodontitis exhibits pathophysiological synergism with cardiovascular disease determinants mediated by circulating mediators including interleukin-6 (IL-6), tumor necrosis factor-alpha (TNF-α), and high-sensitivity C-reactive protein (hsCRP) [9]. Furthermore, it has been recognized by the European Federation of Periodontology and the International Diabetes Federation that periodontitis and diabetes are linked through pathways involving IL-1β, TNF-α, and IL-6, with professional periodontal therapy contributing to a reduction in glycated hemoglobin (HbA1c) levels in diabetic patients [12]. Non-surgical periodontal treatment has also been associated with improvements in systemic inflammation markers and joint disease activity in rheumatoid arthritis patients [13].

### 1.3. Close Relationship Between Periodontitis and Pneumonia

There is ample evidence linking respiratory illnesses, especially pneumonia, to periodontitis [14]. Respiratory pathogens such as *Klebsiella pneumoniae* and *Pseudomonas aeruginosa* can colonize the oral cavity and be aspirated into the lungs, leading to pneumonia [15,16]. Patients with lung diseases exhibit a significantly worse periodontal condition, with the majority of them having a high periodontal index indicating a heightened risk of infection [17]. Studies have demonstrated that patients with moderate to severe periodontitis are four times more likely to experience community-acquired pneumonia (CAP) compared to those without a disease of the periodontal tissues [18]. Moreover, a 7-year longitudinal study revealed that patients with periodontitis undergoing hemodialysis had significantly higher mortality rates from pneumonia than their periodontally healthy counterparts [19]. The oral cavity can serve as a reservoir for respiratory infections, as periodontal disease promotes the accumulation of dental plaque, which fosters the colonization of respiratory pathogens [20,21,22].

This review aims to explore the intricate relationship between periodontitis and systemic health, with a particular focus on its connection to respiratory diseases such as pneumonia, chronic obstructive pulmonary disease (COPD), obstructive sleep apnea (OSA), asthma, lung cancer, and COVID-19. By examining the current literature, we will investigate the role of specific periodontal microorganisms—namely *Porphyromonas gingivalis* (Pg), *Treponema denticola* (TD), *Fusobacterium nucleatum* (Fn), *Aggregatibacter actinomycetemcomitans* (Aa), and *Tannerella forsythia* (Tf)—in influencing respiratory health. These pathogens contribute to the development of respiratory diseases by promoting pathogen adhesion, inducing epithelial cell apoptosis, and triggering inflammatory responses. Furthermore, the review will highlight the potential benefits of periodontal treatment, such as reducing microbial load and systemic inflammation, which may help mitigate the risk of respiratory complications. The importance of incorporating oral health into respiratory disease prevention and therapy is highlighted by this multidisciplinary approach and calls for further research to clarify these associations and develop targeted interventions aimed at improving clinical outcomes.

## 2. Relationship Between Periodontal Microorganisms and Respiratory Health

### 2.1. The Relationship Between Periodontal Microorganisms and Respiratory Diseases

Periodontitis is closely linked to respiratory diseases, including pneumonia. The lungs can be infected by oral pathogens including *Porphyromonas gingivalis* (Pg) and *Fusobacterium nucleatum* (Fn), potentially leading to infections like community-acquired pneumonia (CAP) [23]. In hospital settings, molecular studies of mechanically ventilated patients demonstrate that respiratory pathogens such as *Staphylococcus aureus*, *Pseudomonas aeruginosa*, Acinetobacter species, and enteric species recovered from dental plaque are genetically indistinguishable (>95% similarity by pulsed-field gel electrophoresis) from those isolated from bronchoalveolar lavage fluid, confirming that dental plaque serves as an important reservoir for respiratory pathogens [24]. Molecular evidence further confirms that periodontal biofilms serve as reservoirs for respiratory pathogens. Supporting evidence from institutionalized elders demonstrates this relationship: it was reported that 57% of such patients (28/49) had dental plaques colonized by aerobic pathogens, predominantly *Staphylococcus aureus* (45%), Gram-negative bacilli (42%), and *Pseudomonas aeruginosa* (13%). Crucially, pulsed-field gel electrophoresis (PFGE) genetically matched respiratory pathogens from dental plaques to those causing hospital-acquired pneumonia (HAP) in 8 of 10 microbiologically confirmed cases, establishing the oral cavity as a direct source of respiratory infection in high-risk populations with compromised oral hygiene [16]. These organisms spread from the mouth into the trachea and subsequently into the lung to initiate infection [24].

Patients with periodontal disease, particularly those with weakened immune systems, face an increased risk of respiratory complications [23]. Early detection and management of periodontal disease—through the collaboration between dental and medical professionals—are essential to reducing the risk of pneumonia and improving respiratory health outcomes [23].

Oral pathogens such as *Porphyromonas gingivalis* (Pg), *Fusobacterium nucleatum* (Fn), and chronic obstructive pulmonary disease (COPD), which is linked to periodontal disease, have also been linked to *Aggregatibacter actinomycetemcomitans* (Aa), *Tannerella forsythia* (Tf), and *Treponema denticola* (TD) [25]. Aspiration of these microbes can lead to inflammation and infection in the lungs, and the inflammation caused by periodontal disease may exacerbate COPD symptoms [26]. Additionally, oral dysbiosis exacerbates COPD via the aspiration of pathogens (e.g., Pg and Fn activating lung inflammation), and conversely, systemic inflammation from COPD may disrupt oral microbiota homeostasis [27]. In addition, periodontal disease, dry mouth, and bruxism are frequently linked to obstructive sleep apnea (OSA) [28]. The systemic inflammation caused by OSA can worsen periodontal disease, while mouth breathing exacerbates xerostomia and increases the risk of dental cavities. Patients with OSA, especially those using therapeutic oral appliances for OSA management, require frequent dental check-ups due to the potential changes in dental occlusion [28]. The importance of interdisciplinary care is highlighted, as early dental evaluation and intervention are critical in managing oral health issues associated with OSA [28]. A potential link between periodontal disease and asthma has also been suggested. Asthma may contribute to periodontal disease through the use of inhalers, mouth breathing, and hypoxia [29]. Conversely, the onset and worsening of asthma symptoms may be influenced by periodontal bacteria and the inflammation they cause [29]. According to a number of studies, people with asthma are more likely to acquire periodontal disease, and the association between the two disorders is influenced by common risk factors like smoking and gastroesophageal reflux disease (GERD) [29]. Some research suggests that treating periodontal disease may help alleviate asthma symptoms [29]. Additionally, there might be a link between lung cancer and periodontal disease [25]. Research indicates that a systemic inflammatory response plays a central role in linking periodontal disease to lung cancer. Inflammation caused by periodontal bacteria stimulates the immune system, potentially increasing the risk of lung cancer development [25]. Evidence also suggests that the systemic effects of periodontal bacteria can account for the correlation between periodontal disease and tooth loss and an increased risk of lung cancer. To create a more robust link between periodontal disease and lung cancer, further prospective research is required [25].

**Figure 1:**
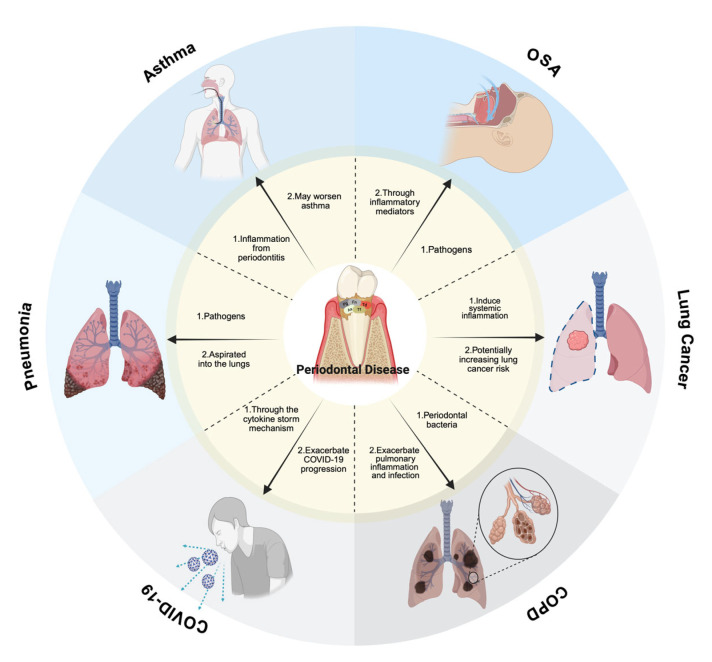
Mechanistic pathways linking periodontal pathogens to respiratory diseases, with oral appliances for OSA treatment specified. The diagram is of the associations with respiratory diseases. Pneumonia: oral pathogens community-acquired pneumonia (CAP) can result by aspirating *Porphyromonas gingivalis* (Pg) and *Fusobacterium nucleatum* (Fn) into the lungs, with immunocompromised individuals at higher risk. COPD: Pg, Fn, *Treponema denticola* (Td), *Aggregatibacter actinomycetemcomitans* (Aa), and *Tannerella forsythia* (Tf) contribute to lung inflammation and infection, exacerbating COPD progression. OSA: periodontal disease, dry mouth, and bruxism are common in OSA patients. Mouth breathing worsens xerostomia and dental caries, while prolonged use of oral appliances may affect occlusion. Asthma: inhaler use, mouth breathing, and hypoxia may worsen periodontitis, while periodontal inflammation may, in turn, exacerbate asthma symptoms. Lung Cancer: periodontal bacteria trigger a systemic inflammatory response, potentially increasing lung cancer risk. An increased incidence of lung cancer is linked to both periodontitis and tooth loss. COVID-19: periodontitis may worsen COVID-19 progression via the cytokine storm mechanism, and maintaining oral health may help reduce respiratory complications. This figure was created with Created in BioRender. Kim, B. (2025) https://BioRender.com/p86m475.

Finally, periodontitis may cause systemic inflammation, which could accelerate the spread of COVID-19. Periodontal disease and COVID-19 are believed to be related through the cytokine storm mechanism [30]. Maintaining proper oral hygiene is crucial in reducing the risk of respiratory complications, though further research is required to fully elucidate this connection [30]. As summarized, emerging evidence suggests periodontal inflammation may exacerbate COVID-19 severity through amplified cytokine release (e.g., IL-6, TNF-α) [30]. Maintaining proper oral hygiene is crucial in reducing the respiratory complications associated with SARS-CoV-2 infection, as depicted in Figure 1’s pathway analysis, which further illustrates the proposed biological pathways linking periodontal dysbiosis to SARS-CoV-2 complications. However, causal mechanisms require validation in large-scale longitudinal studies. The synthesized mechanistic framework of these interactions is presented in Figure 1.

### 2.2. Types and Functions of Periopathogens

**Figure 2:**
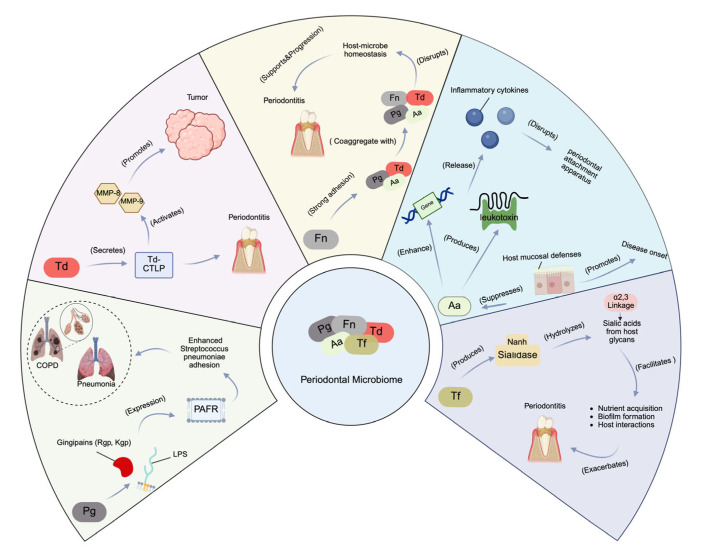
Virulence mechanisms of key periodontal pathogens. Pg: a Gram-negative anaerobe that produces virulence factors like gingipains (Rgp, Kgp), which promote PAFR expression on alveolar epithelial cells, enhancing *Streptococcus pneumoniae* adhesion and potentially contributing to pneumonia. Td: secretes Td-CTLP, a virulence factor linked to periodontitis and orodigestive cancers. Td-CTLP activates MMP-8 and MMP-9, key enzymes in tumor progression. Fn: a pathobiont that coaggregates with Pg, Td, and Aa, facilitating biofilm formation and resisting host defenses. Aa: suppresses host mucosal defenses by enhancing complement resistance genes and leukotoxin production, contributing to immune evasion and periodontal tissue destruction. Tf: produces *NanH* sialidase, which cleaves sialic acids from host glycans, aiding biofilm formation and bacterial–host interactions. This figure was created with Created in BioRender. Kim, B. (2025) https://BioRender.com/7t3mdxt.

*Porphyromonas gingivalis* (Pg), *Treponema denticola* (Td), *Fusobacterium nucleatum* (Fn), *Aggregatibacter actinomycetemcomitans* (Aa), and *Tannerella forsythia* (Tf) are among the many microorganisms that are found in the oral cavity, all of which are implicated in periodontitis [22]. An addition to compromising periodontal health, these microorganisms are implicated in systemic diseases such respiratory problems, rheumatoid arthritis, and cardiovascular disease [31,32]. Patients with chronic periodontitis frequently have saliva that contains *Porphyromonas gingivalis* (Pg), a highly pathogenic, Gram-negative anaerobic bacterium that lives in periodontal pockets. It contains a number of virulence factors, such as fimbriae, gingipains (gingipain protests, GP), and lipopolysaccharide (LPS), which are species-specific trypsin-like proteinases. Kgp targets lysine residues, while Rgp cleaves arginine residues [33]. These gingipains increase the expression of PAFR on human alveolar epithelial cells, which improves *Streptococcus pneumoniae*’s ability to adhere to these cells. This approach raises the possibility that *Porphyromonas gingivalis* (Pg) has a role in pneumonia development [33]. Additionally, Pg’s gingipains (Rgp and Kgp) promote PAFR expression on alveolar cells, enhancing *Streptococcus pneumoniae* adhesion and potentially contributing to pneumonia. *Treponema denticola* (Td) secretes Td-CTLP, a key virulence factor that plays a significant role in periodontitis development [34]. Moreover, Td-CTLP has been detected in various orodigestive cancers, such as esophageal, gastric, and pancreatic tumors, and in vitro studies suggest that it activates matrix metalloproteinases (MMP-8 and MMP-9), critical players in tumor progression [34]. Td-CTLP may contribute to carcinogenesis through immunomodulation. *Fusobacterium nucleatum* (Fn), a Gram-negative, spindle-shaped anaerobe, is one of the most prevalent bacteria in the human oral cavity [35]. It acts as a pathobiont, proliferating during dysbiosis that often precedes periodontal disease, and supports keystone species such as *Porphyromonas gingivalis* (Pg) in disrupting host–microbe homeostasis [35]. *Fusobacterium nucleatum* (Fn) is known for its strong adhesion mechanisms and can coaggregate with early colonizers like Streptococcus species and anaerobic secondary colonizers such as *Porphyromonas gingivalis* (Pg), *Treponema denticola* (Td), and *Aggregatibacter actinomycetemcomitans* (Aa). By linking these species together, Fn plays a critical role in biofilm formation, which resists host defenses like saliva and gingival crevicular fluid [35]. Furthermore, Fn promotes cancer progression by manipulating β-catenin signaling, immune evasion, and chemoresistance, with FadA and LPS playing key roles. *Aggregatibacter actinomycetemcomitans* (Aa) weakens the host’s mucosal defenses, promoting disease onset. A comprehensive review of clinical, molecular, and interventional research that has elucidated the genetic, phenotypic, and biogeographical strategies Aa uses to survive and contribute to disease pathogenesis by suppressing host mucosal defenses can be found at [36]. *Aggregatibacter actinomycetemcomitans* (Aa) can enhance the expression of complement resistance genes and leukotoxin production, thereby modulating the local host immune response to facilitate the overgrowth of a consortium of pathobionts [36]. The consortium collaboratively overcomes the host’s innate immune defenses, triggering the release of inflammatory cytokines. This process ultimately leads to the degradation of connective tissue and bone, resulting in the disruption of the periodontal attachment apparatus [36]. *Tannerella forsythia* (Tf) is an anaerobic bacterium within the “red-complex” of the oral microbiota, which is strongly linked to periodontitis. This bacterium produces sialidases, enzymes that cleave sialic acids from the glycans present on the surface of host cells. Among these sialidases, *NanH* plays a crucial role in *T. forsythia*’s virulence [37]. *Tannerella forsythia* (Tf)’s *NanH* sialidase targets both α2,3 and α2,6-linked sialic acids, preferring the α2,3 linkage, when cleaving sialic acid from host glycans. Beyond aiding in nutrient acquisition, this enzyme facilitates biofilm formation and enhances interactions with host cells by removing sialic acid [37]. Specifically, *NanH* aids nutrient acquisition, promotes biofilm formation, and enhances interactions with host cells by removing sialic acid. Collectively, the virulence mechanisms of these key periodontal pathogens include *Porphyromonas gingivalis* (Pg), *Treponema denticola* (Td), *Fusobacterium nucleatum* (Fn), *Aggregatibacter actinomycetemcomitans* (Aa), and *Tannerella forsythia* (Tf) (Figure 2). 

### 2.3. Interactions Between Periodontal Microorganisms and the Respiratory System: Mechanism Analysis

Periodontal pathogens play a critical role in modulating respiratory health by promoting the adhesion of respiratory pathogens to lung tissues. For example, *Porphyromonas gingivalis* (Pg) upregulates platelet-activating factor receptors (PAFR), which facilitate the attachment of *Streptococcus pneumoniae* to respiratory epithelial cells [32]. Additionally, certain periodontal microorganisms, such as *Fusobacterium nucleatum* (Fn), have been shown to increase ACE2 receptor expression, increasing SARS-CoV-2’s ability to enter host cells during COVID-19 [38,39]. The mechanisms by which periodontal microorganisms affect respiratory health include promoting mucus hypersecretion, inducing apoptosis in lung epithelial cells, and dysregulating immune responses [40,41]. *Fusobacterium nucleatum* (Fn) and *Porphyromonas gingivalis* (Pg) stimulate the production of MUC5AC, leading to mucus overproduction and airway obstruction in diseases such as COPD [40,41]. These pathogens also induce proinflammatory cytokine release, which contributes to the exacerbation of respiratory diseases like pneumonia and COVID-19 [42,43,44].

### 2.4. The Impact of Respiratory Diseases on Periodontal Microbiota

Chronic obstructive pulmonary disease (COPD) and community-acquired pneumonia (CAP) are respiratory illnesses directly associated with the periodontal microbiome. Anaerobic bacteria, commonly found in periodontal disease, have been identified as pathogenic factors in CAP [45]. COPD directly influences periodontal microbiota diversity and abundance. Studies show that patients with both COPD and periodontitis exhibit distinct salivary microbiome profiles compared to those with periodontitis alone or healthy controls [46], characterized by an increased Rothia, Veillonella, and Actinomyces abundance, higher bacterial richness/diversity, and unique Lachnospiraceae prevalence—shifts correlating with aggravated periodontal inflammation suggest that COPD exacerbates oral dysbiosis [46]. Furthermore, the presence of oral pathogens in COPD patients’ sputum and bronchoalveolar lavage fluid confirms the oral microbiota’s crucial role in disease pathogenesis [47]. Pathogens including *Streptococcus pneumoniae*, *Klebsiella pneumoniae*, and *Staphylococcus aureus* colonize dental plaque, periodontal pockets, and saliva, establishing the oral cavity as a significant reservoir for respiratory pathogens [45]. This creates a foundation for lower respiratory infections when host immunity is compromised [45].

**Figure 3:**
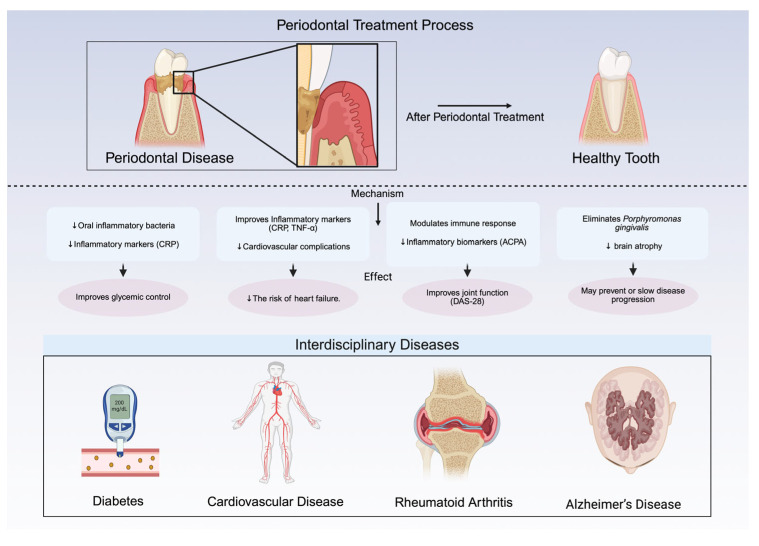
Systemic benefits of periodontal treatment across interdisciplinary diseases. Diabetes: reduces oral inflammation, improves glycemic control, and lowers inflammatory markers like C-reactive protein (CRP); crucial for type 2 diabetes management. Cardiovascular disease (CVD): lowers the risk of myocardial infarction and heart failure by improving inflammatory markers (CRP, TNF-α); particularly beneficial for diabetic patients. Rheumatoid arthritis (RA): reduces inflammation, improves disease activity score 28 (DAS-28), and lowers anti-citrullinated protein antibody (ACPA) levels; interdisciplinary management recommended. Alzheimer’s disease (AD): eliminates *Porphyromonas gingivalis*, slows brain atrophy, and may delay cognitive decline; early intervention is key. This figure was created Created in BioRender. Kim, B. (2025) https://BioRender.com/6lo1gi5.

Crucially, respiratory diseases facilitate the colonization of these pathogens by promoting oral microbiota dysbiosis and impairing immune function. Conditions such as periodontitis, denture stomatitis, and smoking enable colonization, while aging, radiotherapy, chemotherapy, and inhalation therapies (e.g., beta-2 agonists, anticholinergics, and corticosteroids) exacerbate this process [45]. Inhalation therapies alter oral ecology through a reduced salivary flow (diminishing IgA/lysozyme protection) [48], acidic pH shifts promoting cariogenic bacteria [48], and corticosteroid-induced immunosuppression increasing oral candidiasis/ulceration risk [48]. Periodontal pathogens (*Porphyromonas gingivalis*, *Treponema denticola*) prevalent in COVID-19 patients exacerbate respiratory inflammation and enhance SARS-CoV-2 infectivity by degrading its spike protein [49]. They may enter the bloodstream, causing bacteremia and endotoxemia that worsen COVID-19 outcomes [49]. Anatomically, the oral cavity serves as a reservoir for respiratory pathogens due to its connection with the upper respiratory tract [50]. When immunity is compromised (by COPD, smoking, or prolonged hospitalization), oral microbes enter the lungs contributing to respiratory disease development [50]. Collectively, these mechanisms demonstrate that respiratory diseases and their treatments initiate a vicious cycle: COPD/inhalation therapy → oral dysbiosis → pathogen colonization → worsened respiratory disease [46,48]. As illustrated in Figure 3, periodontal treatment provides systemic benefits across various interdisciplinary diseases, including diabetes, cardiovascular disease, rheumatoid arthritis, and Alzheimer’s disease.

## 3. Effects of Periodontal Treatment on Respiratory Health

Periodontal treatment has been shown to reduce the risk of respiratory diseases, particularly in reducing hospital-acquired infections like pneumonia [51]. Professional oral care interventions significantly decrease the oral microbial burden, thereby mitigating the occurrence of ventilator-associated pneumonia among critically ill patients; furthermore, chlorhexidine-based oral rinses have demonstrated efficacy in curtailing respiratory pathogens in the oropharynx, which in turn minimizes the risk of aspiration pneumonia [51]. In individuals suffering from COPD (Chronic Obstructive Pulmonary Disease), periodontal therapy has been linked to improved respiratory function and a reduction in adverse respiratory events [52], and treatment in patients with periodontitis and COPD can slow down lung function deterioration, reduce exacerbation frequency, and improve respiratory function [53]. Patients receiving periodontal treatment exhibit a reduced incidence of respiratory complications—including acute exacerbations, pneumonia, and acute respiratory failure—while COPD patients undergoing periodontal therapy experience fewer visits to medical facilities, hospitalizations, and ICU admissions [52]. Furthermore, compared to the control group, the therapy group’s all-cause death rate is substantially lower [52]. Both COPD and periodontal diseases involve systemic inflammation, and periodontal treatment may help alleviate COPD symptoms by reducing inflammation through the reduction in dental plaque, oral mucosal colonization, and serum inflammatory markers, potentially preventing airway inflammation [52]. Although its influence on quality of life remains uncertain, these findings suggest that periodontal treatment may be beneficial for managing COPD, but additional studies are required to validate these outcomes [53]. Periodontal disease extends beyond the oral cavity and has been linked to a range of systemic conditions, including respiratory diseases [54]. While the exact nature of the causal relationship remains unclear, significant evidence suggests that periodontal treatment can influence the symptoms, events, and biomarkers of several systemic diseases, potentially reducing their severity or prevalence [54]. Recent interventional studies have concentrated on assessing how periodontal treatment influences systemic conditions, with growing recognition of its benefits in managing disorders such as cardiovascular disease, diabetes, and rheumatoid arthritis [54]. Extensive research demonstrates significant beneficial effects beyond the oral cavity: in diabetes, treatment enhances glycemic control and reduces systemic inflammation [55,56,57,58]. For cardiovascular disease (CVD) in periodontitis patients, therapy improves key biomarkers (CRP, TNF-α) and specifically reduces myocardial infarction and heart failure risk in diabetics [59,60,61]. Periodontal therapy also reduces systemic inflammation and improves disease activity scores (DAS-28) in rheumatoid arthritis patients [62,63,64]. Furthermore, evidence links periodontal treatment to potentially slowing Alzheimer’s disease progression, including reduced brain atrophy, suggesting a role in managing early AD risk [65,66]. These results underscore the prospective benefits of periodontal treatment in preventing and managing respiratory health issues, particularly within hospital settings [51].

Daily toothbrushing is a highly effective intervention for reducing hospital-acquired pneumonia (HAP), particularly in critically ill and mechanically ventilated patients, as incorporating it into routine oral care significantly lowers pneumonia incidence and improves critical clinical outcomes, with evidence that brushing reduces plaque, decreases oral pathogens (Pg, Fn), restores the oral microbiome balance, and lowers the frequency of acute COPD exacerbation [27]. Key advantages include its safety profile (no major adverse events reported), feasibility across hospital settings, and equivalence of twice-daily brushing to more frequent regimens, strongly supporting prioritizing it over antiseptic-only protocols in infection prevention guidelines [67]. This is critical because oral hygiene significantly impacts respiratory infection risk in hospitalized patients: Oral microbiota can migrate to the respiratory tract, where specific genera like *Prevotella* (promoting bronchitis) and *Streptococcus pneumoniae* (exacerbating pneumonia) worsen infections; dysbiosis, especially in mechanically ventilated patients, increases ventilator-associated pneumonia (VAP) risk, and enhanced oral care (e.g., antimicrobial rinses, debridement) plus targeted microbiome modulation (e.g., probiotics) effectively reduce respiratory infections in this population [68].

## 4. Impact of Oral Care Interventions on Respiratory Infections in Hospitalized Patients

The oral microbiome, as the body’s second most complex microbial ecosystem, is closely linked to respiratory health through anatomical proximity and immune modulation. A two-sample Mendelian randomization study in an East Asian population [68] confirmed causal associations between specific oral microbial genera and five respiratory infections: *Prevotella* promotes bronchitis but inhibits pneumonia and tonsillitis; *Streptococcus pneumoniae* exacerbates pneumonia, while *Streptococcus pseudopneumoniae* inhibits bronchitis; *Fusobacterium* significantly promotes chronic sinusitis, bronchiectasis, and bronchitis; Pauljensenia and Capnocytophaga are associated with a reduced risk of bronchitis/tonsillitis and inhibited pneumonia progression, respectively, indicating that translocation of oral microbes to the respiratory tract can influence respiratory diseases via the modulation of inflammatory cytokines (e.g., IL-6, IL-8) and enhancement of pathogen virulence. In hospitalized patients, oral hygiene interventions have shown significant preventive effects on respiratory infections: A systematic review and meta-analysis of 15 randomized controlled trials involving 10,742 patients demonstrated that twice-daily mechanical toothbrushing reduced the risk of hospital-acquired pneumonia (HAP) by 33% (RR = 0.67, 95% CI: 0.56–0.81) [67], with similar benefits in mechanically ventilated patients (RR = 0.68, 95% CI: 0.57–0.82) [67], and no major adverse events reported; the intervention also decreased ICU mortality by 19% (RR = 0.81, 95% CI: 0.69–0.95) [67], shortened the duration of mechanical ventilation by 1.24 days (95% CI: −2.42 to −0.06) [67], and reduced the ICU length of stay by 1.78 days (95% CI: −2.85 to −0.70) [67]. Because twice-daily brushing was as effective as more frequent regimens and far superior to antiseptic-only protocols, mechanical oral cleaning interventions should be prioritized in infection prevention guidelines [67]. Combined strategies—antiseptic rinses, mechanical debridement, and targeted microbiome modulation (e.g., probiotics)—effectively reduce respiratory infections in hospitalized, particularly mechanically ventilated, patients, improving clinical outcomes and survival [67,68].

## 5. Recent Progress in the Study of Periodontal Microorganisms and Respiratory Health

### 5.1. New Technologies and Methods for Periodontal Microbiome Research

Recent progress in high-throughput sequencing and metagenomic analysis has transformed the study of the periodontal microbiome, enabling deeper insights into microbial diversity and function [69,70]. Complementing these modern approaches, traditional molecular typing techniques like pulsed-field gel electrophoresis and multilocus sequence typing remain vital for determining the genetic relatedness of microbial strains in clinical settings [16]. These technologies provide researchers with the means to investigate the interactions between periodontal pathogens and respiratory health at an unprecedented level of detail.

### 5.2. New Discoveries and Theoretical Breakthroughs in the Field of Respiratory Health

Recent discoveries have underscored how periodontal pathogens contribute to the entry and replication of respiratory viruses, including SARS-CoV-2 [38,39]. These findings have significant implications for understanding how oral health influences respiratory health amid viral infections such as COVID-19. Beyond viral infections, an HIV study demonstrates that oral dysbiosis correlates with impaired pulmonary function and systemic inflammation, suggesting oral microbiota as a biomarker for respiratory outcomes in comorbid lung diseases [27].

### 5.3. Frontier Trends in Interdisciplinary Research

The integration of periodontal and respiratory health into interdisciplinary care models is an emerging trend in medical research. Studies are increasingly recognizing the importance of addressing the oral–pulmonary axis to improve overall patient outcomes. Interdisciplinary approaches that involve the collaboration between dental and medical professionals hold promise for enhancing the management of both periodontal and respiratory diseases [42,43].

## 6. Challenges and Prospects

### 6.1. Limitations of Periodontal Microorganisms and Respiratory Health Research

Even while there is mounting evidence linking periodontal microbes to poor respiratory health, several limitations continue to challenge the field. Many studies are constrained by small sample sizes, which limit their generalizability across larger populations [71]. Furthermore, the diagnostic criteria for both periodontitis and respiratory diseases are often inconsistent, complicating efforts to standardize findings and make cross-study comparisons [71].

Another significant limitation is the predominance of cross-sectional studies, which can only establish correlations rather than causality between periodontal disease and respiratory conditions. Equally important is the unresolved debate regarding directionality (oral → lung vs. lung → oral), particularly the lack of direct evidence on how COPD specifically alters periodontal microbiota, emphasizing the need for longitudinal studies on respiratory disease-induced oral dysbiosis [27]. Longitudinal and interventional studies are essential to gain a clearer understanding of the relationship between these conditions over time [72].

In addition, while most research focuses on bacterial pathogens, the potential role of viruses, fungi, and other microorganisms in the oral–respiratory axis remains underexplored. This gap leaves much to be discovered about the full spectrum of microbial involvement in respiratory health. Furthermore, confounding factors such as smoking, immunosuppressive therapy, and systemic conditions like diabetes are not always adequately accounted for, introducing potential bias [73].

The lack of randomized controlled studies (RCTs) examining how periodontal care affects respiratory outcomes is another drawback. Most of the current literature focuses on observational data, which does not allow for definitive conclusions regarding the impact of treating periodontitis on lung health [72].

### 6.2. Future Research Directions and Suggestions

To address these challenges, future research should prioritize the design and implementation of well-powered longitudinal cohort studies and interventional studies that utilize consistent diagnostic criteria. Such studies will help to establish stronger causal links between periodontitis and respiratory diseases. Moreover, it will be critical to incorporate standardized assessments for oral and respiratory health, alongside a comprehensive control for confounding variables like smoking and medication use [72].

There is also a pressing need to expand research beyond bacterial pathogens to explore the roles of viruses, fungi, and polymicrobial interactions in the oral–respiratory axis. Advanced microbiome analysis techniques, such as metagenomics and multi-omics approaches, should be employed to deepen our understanding of the interactions between oral pathogens and the host immune system [72,74,75]. This will enhance our ability to investigate how periodontal pathogens contribute to respiratory diseases like pneumonia, asthma, and COPD.

Future research should also investigate the use of probiotics, prebiotics, and other microbial interventions to restore balance within the oral microbiome and potentially improve respiratory health outcomes [76,77]. Additionally, identifying specific biomarkers that predict the development of respiratory disease based on the oral microbiome composition could open up new avenues for early diagnosis and preventive care [78,79,80]. Critically, research insights must translate into clinical practice, particularly in high-risk settings. Current oral care in institutional settings often remains inadequate, with healthcare providers in these facilities rarely assisting residents with tooth/denture cleaning—even when trained. Implementing evidence-based, structured oral care programs (e.g., professional weekly cleaning combined with post-meal brushing) are essential, as such interventions have been shown to significantly reduce pneumonia-related mortality [16].

Finally, interdisciplinary collaboration between dentists, pulmonologists, and infectious disease specialists will be critical for advancing this field. Such cooperation will foster the development of novel treatment strategies targeting both oral and respiratory health [52,77,80,81,82].

## 7. Conclusions

It is commonly known that periodontitis is a major risk factor for a number of respiratory conditions, such as pneumonia and Chronic Obstructive Pulmonary Disease (COPD), Obstructive Sleep Apnea (OSA), asthma, lung cancer, and COVID-19. Periodontal pathogens such as *Porphyromonas gingivalis* (Pg), *Treponema denticola* (Td), *Fusobacterium nucleatum* (Fn), *Aggregatibacter actinomycetemcomitans* (Aa), and *Tannerella forsythia* (Tf) play a crucial role in respiratory diseases by promoting pathogen adhesion, inducing epithelial cell apoptosis, and triggering inflammatory responses. Molecular studies definitively identify dental plaque colonizers as key reservoirs for hospital-acquired pneumonia (HAP) pathogens in hospitalized elders. Consequently, integrating routine oral hygiene into respiratory health protocols is imperative for mitigating infection risks in vulnerable populations.

In addition to respiratory diseases, periodontal disease is closely associated with systemic conditions, including diabetes, cardiovascular diseases, rheumatoid arthritis, and Alzheimer’s disease. By intensifying systemic inflammation, these infections may aid in the development of various illnesses.

Although significant evidence supports these associations, more large-scale, rigorous studies are needed to establish definitive causality. The current limitations primarily stem from the lack of high-quality Randomized Controlled Trials (RCTs) and longitudinal studies, which are essential for establishing causal links between periodontal disease and respiratory health outcomes. Future research should prioritize designing and implementing high-quality clinical trials with long-term follow-up to establish clearer causal relationships. Moreover, research should expand beyond bacterial pathogens to include other microorganisms, such as viruses and fungi, to provide a more comprehensive understanding of the oral–respiratory axis.

An interdisciplinary approach is crucial, as this review emphasizes, particularly in integrating oral health as a critical component in managing and preventing respiratory diseases. Periodontal treatment, by reducing the microbial burden and systemic inflammation, may effectively alleviate symptoms and reduce the occurrence of respiratory complications. This interdisciplinary approach not only promotes collaboration between dental and medical fields but also offers a new perspective on integrating oral health into overall health management. Integrating the gut–oral–lung axis framework further supports that oral microbiome modulation (e.g., via statins or hygiene) may improve COPD outcomes, while acknowledging bidirectional influences between respiratory and periodontal health [27].

Additionally, periodontal treatment shows potential benefits in managing systemic diseases, including diabetes, cardiovascular diseases, rheumatoid arthritis, and Alzheimer’s disease. Studies suggest that periodontal treatment can improve glycemic control in diabetic patients, reduce cardiovascular risk, lower disease activity in rheumatoid arthritis, and delay the progression of Alzheimer’s disease. Early screening and treatment of periodontal disease, particularly in high-risk groups such as COPD and diabetes patients, could serve as an effective strategy to reduce respiratory complications. Therefore, we advocate for incorporating oral health into comprehensive health management in clinical practice, particularly in the prevention and treatment of respiratory diseases, diabetes, cardiovascular diseases, rheumatoid arthritis, and Alzheimer’s disease, with future studies expected to provide more evidence to support this field.

## Abbreviations

Aa	*Aggregatibacter actinomycetemcomitans*
ACPA	Anti-Citrullinated Protein Antibodies
AD	Alzheimer’s Disease
CAP	Community-Acquired Pneumonia
COPD	Chronic Obstructive Pulmonary Disease
CRP	C-Reactive Protein
CVD	Cardiovascular Disease
DAS-28	Disease Activity Score 28
Fn	*Fusobacterium nucleatum*
GERD	Gastroesophageal Reflux Disease
HbA1c	Glycated Hemoglobin A1C
ICU	Intensive Care Unit
IL-1β	Interleukin-1 Beta
IL-6	Interleukin-6
Kgp	Lysine-specific Gingipain
LPS	Lipopolysaccharide
MMP-8	Matrix Metalloproteinase-8
MMP-9	Matrix Metalloproteinase-9
MUC5AC	Mucin 5AC
OSA	Obstructive Sleep Apnea
PAFR	Platelet-Activating Factor Receptor
Pg	*Porphyromonas gingivalis*
Ra	Rheumatoid Arthritis
RCTs	Randomized Controlled Trials
Rgp	Arginine-specific Gingipain
Td	*Treponema denticola*
Td-CTLP	*Treponema denticola* Chymotrypsin-Like Proteinase
Tf	*Tannerella forsythia*
TNF-α	Tumor Necrosis Factor-Alpha
